# Acute Carbamazepine Intoxication

**DOI:** 10.3390/neurolint14030049

**Published:** 2022-07-22

**Authors:** María Dolores Calabria Gallego, Mónica Alañá García

**Affiliations:** 1 IBSAL, Institute for Biomedical Research of Salamanca, 37007 Salamanca, Spain; 2 Neurology Department, Universitary Hospital of Salamanca, 37007 Salamanca, Spain; malana@saludcastillayleon.es

**Keywords:** carbamazepine, acute intoxication, neurological side effects

## Abstract

Carbamazepine is an anticonvulsant drug with multiple mechanisms of action, which condition the presence of a characteristic clinical picture after the overingestion of the drug. We expose a case report about a patient who, in the context of an attempted suicide, presented acute intoxication by benzodiazepines and carbamazepine, presenting the characteristic clinical picture of fluctuations in the level of consciousness, even presenting gaze deconjugation, almost unreactive coma and generalized hypotonia.

## 1. Introduction

Carbamazepine is an anticonvulsant drug with a wide spectrum of possible therapeutic effects (management of: bipolar disorder [1], impulse control disorder [2], trigeminal neuralgia [3], among others), prescribed by general physicians, psychiatrists, neurologists,… Its mechanism of action is related to the blockade of presynaptic voltage—dependent sodium channels [4], leading consequently to inhibition of the release of synaptic glutamate and possibly other neurotransmitters [5]. Furthermore, carbamazepine is also a potent inhibitor of acetylcholine receptors, adenosine receptors, and N-methyl-D aspartate (NMDA) receptors [6]. These mechanisms of action will be involved in the possible side effects derived from carbamazepine intoxication.

Carbamazepine is metabolized in the liver where the epoxide pathway is the most important, giving the 10,11-transdiol derivative and its glucuronide as the main metabolites [7].

The most commonly observed effects after acute overdose of this drug are ataxia and nystagmus. The presence of cardiotoxicity is also frequent, and an electrocardiogram is mandatory when it exists a suspicion of the mentioned overdose. Depending on the concentration of carbamazepine in blood, some clinical effects or others will be more frequent (Table 1) [8].

However, the effect of exposure to other central-nervous-system (CNS) acting medications on the neurological manifestations of carbamazepine toxicity has not widely been evaluated; this practice is especially frequent when attempts of suicide are committed [9]. In the past, we can find reports of patients who who ingested large quantities of carbamazepine with alcohol and/or other anticonvulsants. They usually presented obtunded with ataxia, nystagmus, mydriasis, nausea and vomiting. They often manifested hypertonicity, hyper-reflexia and clonus early with hypotonia [10].

It can be difficult to identify this diagnosis; due to other many entities can cause the same neurological effects (Table 2), being one of these, just the one for which this drug is prescribe: seizures.

## 2. Case Report

This patient, with personal history of eosinophilic colitis, asthma, and under follow-up in a neurology clinic for long-standing partial epilepsy, treated with carbamazepine, and seizure-free for 5 years, with previous seizures consisting of episodes of staring and disconnection from the environment, sometimes with clonic movements of the extremities. In addition, the patient presented a possible generalized anxiety disorder, for which she had not yet been evaluated by a Mental Health team, although she was being treated for this reason with Alprazolam by her general practitioner. There were no other medical records of interest.

The patient was taken to the Emergency Department by her relatives when she was found fallen, between the bedside table and the bed, with a low level of consciousness, although presenting fluctuations that allowed a coherent language on the way from her home to the hospital. In that moment she confessed have ingested 6 alprazolam tablets. The patient’s relatives found, in fact, an empty blister pack of that drug in the garbage container.

Once in the Emergency Department, two ampoules of intravenous flumazenil were administered to reverse the possible effect of a benzodiazepine intoxication, but as the condition did not improve, it was decided to contact Neurology on call.

The patient was afebrile, with blood pressure of 151/84 mmHg, partial oxygen saturation of 100%, and capillary blood glucose of 198 mg/dL. As for the general examination, she was eupneic, with normal coloration of the skin and mucous membranes, and without signs suggestive of heart failure. On cardiac auscultation, the patient was rhythmic and without murmurs. Pulmonary auscultation in anterior planes showed preserved vesicular murmur, although at times it suggested the presence of hypoventilation. On abdominal palpation, a bladder balloon was detected. There were no edemas, nor signs of deep vein thrombosis.

## 3. Results

On neurological examination, the patient was found alternating between almost unreactive coma with deconjugated pupils and generalized hypotonia, and an alert level of consciousness with significant psychomotor agitation that required of mechanical restraint.

**Figure 1:**
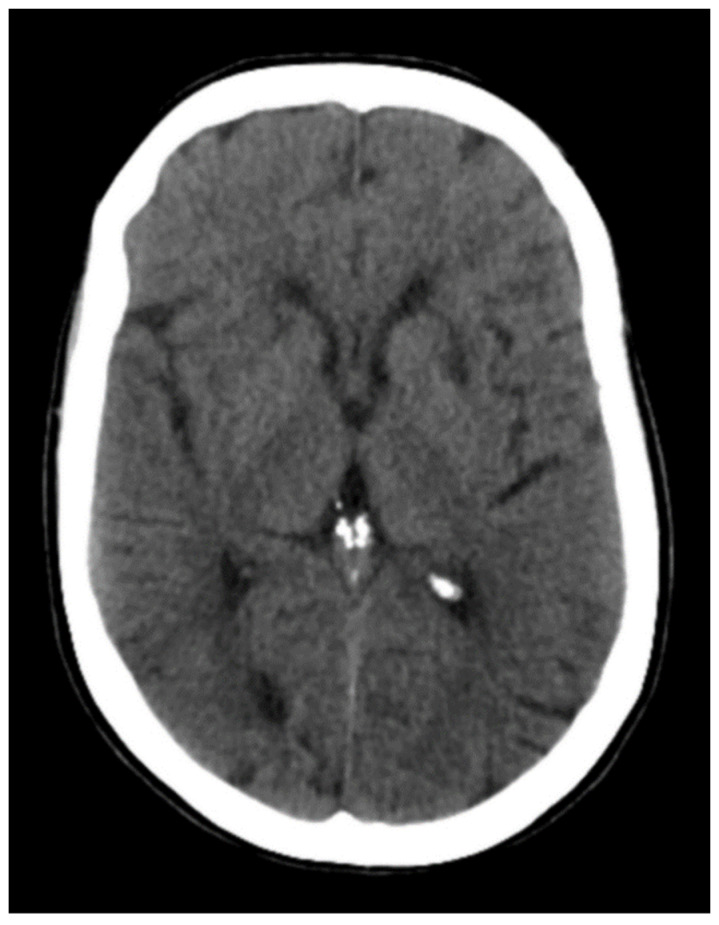
No intra- or extra-axial bleeding signs of acute ischemia (ASPECTS 10) or occupying lesions were observed in the simple cranial CT.

**Figure 2:**
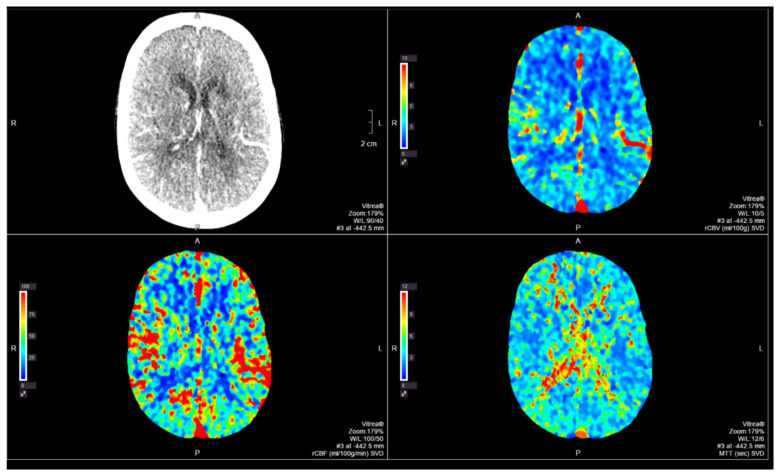
No alterations are observed in the parametric maps (mean transit time, flow or brain volume) suggesting acute cerebral ischemia.

Multimodal cranial computerized tomography (CT) with perfusion sequence was performed (Figure 1 and Figure 2), without alterations; as well as an extensive analysis that showed no alterations of interest, except for the aforementioned hyperglycemia. Levels of toxic substances were requested, detecting high levels of benzodiazepines (>900 mg/dL) and carbamazepine (46.3 mg/dL) (chemiluminescent immunoassay technique with double checked, was performed). Gastric lavage or activated charcoal administration were not performed due to the long period of time between the possible intake of the drug and the knowledge of the carbamazepine values (more than 8 h).

The patient was transferred to the Intensive Care Unit and prescribed support treatment. One day later, she was transferred to a conventional hospital room, with a good level of consciousness and normal neurological status, being able to self-criticize the facts, and confessing the intake of benzodiazepines and carbamazepine for autolytic purposes at a moment when she did not feel able to handle the psychological stress caused by her family situation. The patient is discharged, with outpatient follow-up by Psychiatry Service, not detecting a psychiatric disorder, but remaining from now on in follow-up by a clinical psychologist to better manage the stress caused by her family situation.

## 4. Discussion

Acute intoxication by carbamazepine at high doses is a rare clinical event, being more common the presence of intoxications with other types of toxic substances such as benzodiazepines [12] or alcohol [13,14], however, in a patient with an abnormal neurological situation and with concomitant antiepileptic drugs use, these should be monitored, given the wide availability to determine the levels of them in plasma, so that scenarios like the one that encompasses us do not go unnoticed, and which may require specific urgent management.

As we know intoxication depends on both pharmacodynamics and pharmacokinetics of each drug, we must act in both spheres.

In general, if too much time has not passed since carbamazepine was ingested (no longer than that corresponding to its digestive absorption), treatment with activated charcoal is recommended. Multiple-dose activated charcoal is more efficient [15] than simple-dose; it permits a constant decrease of the half-life of blood CBZ without any rebound effect and could improve the prognosis by reducing the duration of coma and the length of stay.

Another therapeutic option, although not performed in the case report we present, would be the combination of continuous renal replacement therapy with hemoperfusion, which can be easily deployed, appears safe, and is able to combine the carbamazepine mass removal achieved with each technique, thus to maximize carbamazepine extraction [16].

In these cases, above all, the control of possible alterations in the cardiological sphere is of paramount importance. Two major clinical pictures can occur in this regard: sinus tachycardias in the setting of a massive carbamazepine overdose, and the other one is restricted almost exclusively of elderly women who developes potentially life-threatening bradyarrhythmias or atrioventricular conduction delay, associated with either therapeutic or modestly elevated carbamazepine serum levels [17]. Prompt management of both tables is crucial.

No less important will be to carry out an adequate assessment and follow-up in the psychiatric sphere, when the intoxication has been motivated by suicidal purposes.

Finally, although it has been reported infrequently, one of the effects of carbamazepine intoxication to be taken into account is the appearance of hyperglycemia, such as the one that can be seen in the case report at hand [18].

## Figures and Tables

**Table 1:** Correlation between plasma concentrations of carbamazepine and clinical consequences.

Plasma Concentration	Clinical Expression
4–12 mcg/mL	Therapeutic range
12–16 mcg/mL	Ataxia, nystagmus
16–24 mcg/mL	Obtundation, stupor
24–40 mcg/mL	Alternating between reactive coma, agitation, hallucinations, choreographic movements
40–60 mcg/mL	Reactive coma, mydriasis, risk of seizures, decerebrate movements
>60 mcg/mL	Unreactive coma, hypoventilation, cardiotoxicity, possible status epilepticus.

**Table 2:** Main causes of sudden onset coma [11].

Cause	Type
Stroke	Ischemic, hemorrhagic
Seizure	Including non-convulsive status epilepticus
Drug overdose/toxins	Opioids, CO, antipsychotics, antidepressants, antiepileptic drugs…
Metabolic	Hypoglycaemia, hyponatraemia, hypercalcaemia, hyperammonaemia, hepatic encephalopathy…
Infection	Including central nervous system infections

## Data Availability

Not applicable.
